# Bionic Intelligent Interaction Helmet: A Multifunctional-Design Anxiety-Alleviation Device Controlled by STM32

**DOI:** 10.3390/s25103100

**Published:** 2025-05-14

**Authors:** Chuanwen Luo, Yang You, Yan Zhang, Bo Zhang, Ning Li, Hao Pan, Xinyang Zhang, Chenlong Wang, Xiaobo Wang

**Affiliations:** 1 Department of Architecture, School of Architecture and Art, North China University of China, Jinyuanzhuang Road 5, Shijingshan District, Beijing 100144, China; youyang@mail.ncut.edu.cn (Y.Y.); zy2021@mail.ncut.edu.cn (Y.Z.); abaodoc@ncut.edu.cn (B.Z.); panhao@mail.ncut.edu.cn (H.P.); xinyangzhang@mail.ncut.edu.cn (X.Z.); wangchenlong@mail.ncut.edu.cn (C.W.); wangxiaobo@ncut.edu.cn (X.W.); 2 Beijing Historical Building Protection Engineering Technology Research Center, Beijing University of Technology, Beijing 100124, China; ning_li11@bjut.edu.cn

**Keywords:** bionic intelligent interaction helmet, STM32, human–machine interactions

## Abstract

Due to accelerated urbanization, modern urban residents are facing increasing life pressures. Many citizens are experiencing situational aversion in daily commuting, and the deterioration in the traffic environment has led to psychological distress of varying degrees among urban dwellers. Cyclists, who account for about 7% of urban commuters, lack a sense of belonging in the urban space and experience significant deficiencies in the corresponding urban infrastructure, which causes more people to face significant barriers to choosing cycling as a mode of transportation. To address the aforementioned issues, this study proposes a bionic intelligent interaction helmet (BIIH) designed and validated based on the principles of bionics, which has undergone morphological design and structural validation. Constructed around the STM32-embedded development board, the BIIH is an integrated smart cycling helmet engineered to perceive environmental conditions and enable both human–machine interactions and environment–machine interactions. The system incorporates an array of sophisticated electronic components, including temperature and humidity sensors; ultrasonic sensors; ambient light sensors; voice recognition modules; cooling fans; LED indicators; and OLED displays. Additionally, the device is equipped with a mobile power supply, enhancing its portability and ensuring operational efficacy under dynamic conditions. Compared with conventional helmets designed for analogous purposes, the BIIH offers four distinct advantages. Firstly, it enhances the wearer’s environmental perception, thereby improving safety during operation. Secondly, it incorporates a real-time interaction function that optimizes the cycling experience while mitigating psychological stress. Thirdly, validated through bionic design principles, the BIIH exhibits increased specific stiffness, enhancing its structural integrity. Finally, the device’s integrated power and storage capabilities render it portable, autonomous, and adaptable, facilitating iterative improvements and fostering self-sustained development. Collectively, these features establish the BIIH as a methodological and technical foundation for exploring novel research scenarios and prospective applications.

## 1. Introduction

### 1.1. Urban Commuting and Psychological Well-Being

With the continuous acceleration of urbanization and improvements to transportation infrastructure, over half of the world’s population now resides in urban areas and the proportion is projected to reach 70% by 2050 [1]. In particular, the urban population in Asia is expected to reach 2.6 billion [2,3]. Urban commuting is closely related to social, political, economic, and environmental development [4]. Urbanization not only escalates transportation demand and expands transportation networks but also affects economic activities and accessibility while fostering energy-intensive activities [5]. Meanwhile, substantial evidence indicates that urban design and travel patterns influence environmental exposure and lifestyle, both of which are directly correlated with the incidence and mortality rates of urban populations [6]. Rapid urban growth has exacerbated urban transportation challenges, including increased traffic congestion, exhaust pollution, and greenhouse gas emissions, which severely harm the health of urban residents [7,8,9], becoming a serious public safety issue. This leads to sleep disorders, cardiovascular diseases, stress, and premature death among urban populations [10,11,12]. Inadequate urban and transportation planning has a direct impact on over 20 million people with traffic congestion [13], and approximately 82 million people are exposed to harmful levels of urban traffic noise (Lden ≥ 55 dB) daily [14]. Moreover, nearly 3000 premature deaths, accounting for 20% of total deaths, and 50,000 disability-adjusted life years occur annually [13,15,16,17,18]. Through urban planning adjustments (such as the addition of rail transit and optimization of work–residence balance), the traffic congestion index in some U.S. and European cities has decreased by approximately 15–20% since 2015, with a gradual reduction in the affected population [13,19].

Correspondingly, the psychological well-being of people during transportation has gradually become a focus. In fast-paced urban transportation, many individuals face varying degrees of psychological distress, such as anxiety, stress, and loneliness, which may lead to a series of psychological problems. These problems not only affect the quality of life of individuals but also have specific negative impacts on the overall functioning of society [20]. Situational aversion, as a strong negative emotion and avoidance behavior triggered by specific situational stimuli, often originates from adverse events or unpleasant experiences that individuals have encountered in certain situations [21]. Individuals build a conditioned reflex during daily urban travel, such as situational aversion, between negative feelings and scenarios such as vehicle scrapes, frequent traffic congestion, and traffic noise. When experiencing the same scenarios, this reflex leads to aversion or escape emotions to ease the negative feelings [22]. This has been widely of concern by researchers in understanding the relationship between human emotions and behaviors. Currently, research reveals that approximately 55% of the global population resides in urban areas, with 1.78 billion individuals experiencing psychological distress caused by transportation. Around 7% of urban commutes worldwide are made on bicycles, 30% of which have experienced at least one traffic accident in the past year, and 60% of them face the prominent issue of daily frequent safety hazards [23,24,25,26,27].

Urban cycling, as an environmentally friendly, healthy, and convenient mode of transportation, is transforming people’s lives. While cycling presents fewer operational complexities than motor vehicles or public transportation systems, the majority of urban cyclists nevertheless encounter substantial challenges during their daily commutes: limited urban road space, mixed traffic with vehicles, motors, non-motorized vehicles, and pedestrians, yet with incomplete or even absent urban bicycle lane systems [4,28,29,30,31,32]. Cyclists are forced to ride on motor vehicle lanes or sidewalks, competing for road space with motor vehicles and electric scooters, which increases the risk of collisions with motor vehicles and keeps cyclists in a constant state of tension, severely lacking a sense of spatial belonging in urban areas. The complex traffic environment brings safety hazards and requires high levels of physical and mental investment. These stressors, caused by various factors, may trigger stress responses in cyclists [33,34]. When cyclists are in stressful situations for extended or frequent periods, they may develop anticipated traffic anxiety regarding potential traffic conditions, with the continuous accumulation of negative emotions that the fast-paced urban lifestyle cannot alleviate. At this point, cyclists may try to avoid riding on roads as much as possible. Even without the presence of stressors, just mentioning the scenario of road cycling can evoke aversion, leading to a strong aversion to road cycling in the future. This reduces people’s desire to choose cycling as a healthy and efficient mode of transportation, which is not conducive to the development of healthy and sustainable cities. Guided by this current situation, people are beginning to seek ways and means to alleviate this psychological stress in order to improve the overall traffic environment, enhance daily travel experiences, relieve stress, boost brain function, and increase happiness and well-being.

### 1.2. Literature Review

With urbanization, the pace of life has significantly accelerated, work pressures have increased, and the cost of living has risen, especially in expenditures related to housing, education, and healthcare. This high-intensity lifestyle keeps urban residents in a state of chronic stress, making them prone to psychological problems such as anxiety and depression.

#### 1.2.1. The Impact of Urban Diseases and Urban Traffic on Psychological Stress

With the rapid development of urbanization driven by industrialization, a series of problems have emerged, such as population congestion, housing shortages, and environmental pollution. The term “Urban Disease” originated in this period and has gradually become well known. It refers to the phenomenon of low overall urban operational efficiency, disordered urban functional systems, malfunctioning agglomeration and diffusion functions, and decreased residents’ sense of well-being due to improper resource allocation. Specifically, it manifests as traffic congestion, housing shortages, insufficient water supply, energy scarcity, environmental pollution, and disorderly urban conditions [35]. Mark J. Nieuwenhuijsen’s systemic planning of urban transportation provides necessary references for alleviating urban diseases such as traffic congestion and population expansion [15,36]. Omonov’s application of intelligent transportation system technology offers new insights and directions for addressing population expansion in large cities [5].

Urban transportation issues are not only a significant manifestation of urban diseases but also a catalyzer of other urban diseases, to some extent. Urban transportation and urban diseases influence each other. The development of urban transportation can both alleviate and exacerbate urban diseases. Traffic congestion, air pollution, and energy consumption increase the risks to people’s physical and mental health, leading to an increasing number of people in cities suffering from mental illnesses, such as mania, anxiety disorder, phobic neurosis, obsessive–compulsive disorder, and hypochondriasis. Studies have confirmed that these diseases are directly or indirectly related to the problems existing in urban construction [37,38,39,40]. A well-developed built environment and comprehensive facilities can encourage people to choose more sustainable travel modes, such as walking, cycling, and public transportation, thereby reducing traffic congestion, air pollution, and energy consumption. Also, the well-developed transportation system is a crucial factor influencing travel mode choices, and an increase in neighborhood population density and land-use mix can suppress the use of private cars by high-income groups. To mitigate the negative psychological impacts of urban diseases, a comprehensive intervention approach is required, encompassing environmental modification, personal protection, behavioral adjustment, and policy support [32,41]. Avila et al. found that cycling commuting was inversely related to perceived stress [30,42]. Alimohammadi et al.’s cognitive study in a road traffic noise environment (71 dBA) revealed that noise increased the percentage of correct answers but had no effect on response speed [43,44,45]. These findings highlight the urgency of improving urban traffic conditions and the necessity of reducing congestion and commuting time for mental health [46,47]. By planning traffic routes rationally, developing public transportation, and encouraging green travel, the travel efficiency of residents can be improved, and the negative psychological impact of traffic stress can be reduced [48,49].

#### 1.2.2. Research on Relieving Urban Diseases and Psychological Stress

Sociologists have defined and analyzed psychological stress in urban diseases from different aspects. Eunae Jin et al. argued that time pressure and uncertainty caused by traffic congestion, stress from social comparison and competition, and stress from environmental factors were all significant sources of psychological stress in urban diseases [50]. Jenny et al. advocated for therapeutic urbanism, emphasizing the alleviation of stress through urban design. Their study on the therapeutic community project in London found a 19% decrease in the incidence of depression among residents [51]. Thomas Papa et al. proposed the concept of a 15 min city, suggesting that reducing the radius of residents’ daily activities can decrease reliance on transportation and thereby improve psychological load [52]. Their empirical study showed that after the pilot in Copenhagen, the incidence of depressive symptoms among residents decreased by 9.5% [53]. M. Angelidou proposed that optimizing public transportation accessibility, such as increasing subway frequencies and expanding bike-sharing networks, could significantly reduce commuters’ anxiety levels. The team’s tracking data from multiple European cities showed that a 10% increase in public transportation efficiency led to a 7.2% decrease in perceived commuting stress among residents [54].

Research on psychological stress in urban diseases focuses on identifying its causes and providing therapeutic recommendations. They generally agree that psychological stress in urban diseases stems from work pressure, life pressure, and interpersonal relationships [55]. Mary et al. suggested that mental health interventions and education should be implemented, recommending cognitive–behavioral therapy to help individuals manage traffic anxiety, which could reduce anxiety levels by 30–40%. By enhancing emotional awareness and reducing cortisol levels, mindfulness meditation has been validated to decrease anxiety scores by more than 25% after an 8-week training period [56]. Kurtses Gürsoy B’s team proposed that biofeedback and digital interventions could be adopted to treat psychological stress. Clinical data indicated that these methods could improve sleep quality and reduce headache frequency by 40%. Mobile health applications based on big data provided personalized suggestions and have been shown to reduce stress levels by 23% in pilot studies [57].

Similarly, scholars of transportation and urban planning, such as Jessica et al., have systematically studied psychological stress in urban diseases, focusing on the impact of structural factors such as traffic congestion and imbalanced work–residence spaces on residents’ mental health. They proposed multidimensional intervention strategies. Their research found that extended commuting times due to traffic congestion significantly increase residents’ anxiety levels, with a positive correlation between stress indices and congestion duration, with a correlation coefficient of 0.67, *p* < 0.01 [58,59,60]. Stefan Gossling’s team suggested that greenway systems and pedestrian networks could alleviate psychological stress. Taking Copenhagen as an example, they found that bicycle-friendly urban planning increased residents’ contact with nature by 40% and significantly improved emotional stability by reducing traffic noise pollution [61]. Anne Cleary’s team proposed that urban planning should also consider residents’ subjective perceptions of green spaces, suggesting that improving these perceptions could enhance psychological well-being, which was directly related to the improvement of psychological welfare [62].

#### 1.2.3. Relevant Foundations of Bionics Design

Bionics is an interdisciplinary domain that studies the structure, function, and behavior of biological systems and applies these principles to engineering and technology. Bionics design solves complex engineering problems by imitating the structure, function, and behavior of biological entities in nature. Its core lies in the innovative path of learning from nature. The application of bionics design in aspects such as color, structure, texture, form, and function provide various forms and creative spaces for different types of design [63,64,65]. In 1800, the British individual Kelly mimicked the shape of a trout to propose a streamlined structure [66]. In 1870, the German Lilienthal drew inspiration from the flight of storks and developed a curved, ribbed monoplane glider [67]. In 1959, the term *bionics* first appeared in 1959, originating from the combination of the Greek prefix *bio-* (meaning life) and the word *electronics*. This term was initially coined by American aerospace engineer Jack E. Steele in his 1957 publication, *Man and Space* [68].

In recent decades, bionics has achieved significant development. As an interdisciplinary research area, it has garnered increasing attention. Numerous researchers have conducted extensive bionics design studies across various fields and proposed a series of valuable theories. Researchers applied bionics principles to conduct a bionics design, and found that various organisms, to survive in nature, adapted to their surroundings during the evolutionary process, gradually changing their forms to develop various scientifically sound structural configurations. In the architecture discipline, by studying the relationship between the natural distribution characteristics of bamboo structural features and their load-bearing characteristics, a bionics strategy for optimizing the stability of regular box girder stiffeners with variable spacing was established [69,70,71]. The number of stiffeners was reduced from 15 to 10, the weight of the two main beams was reduced by 136.12 kg, and the differences in buckling resistance and anti-instability capabilities of various sections were minimized, while still meeting the design requirements for strength and stiffness. A particular study investigated the bionics design method for wind turbine tower cylinders and creatively proposed the bionic-bamboo tube (BBT). The maximum deformation and maximum stress were reduced by 5.93% and 13.75%, respectively, while the natural frequency and overall stability were increased by 3% and 1.1%, respectively [68].

Integrating bionic into design offers numerous benefits. It draws inspiration from natural organisms, enabling designs to conform to natural laws, thereby enhancing the functionality and efficiency of products. Moreover, it endows products with unique appearances and aesthetic appeal, increasing their attractiveness. Meanwhile, by leveraging the adaptability of organisms, it aligns designs more closely with environmental demands, facilitating sustainable development and reducing energy consumption and resource waste. This ultimately creates higher-quality, environmentally friendly, and more efficient products and environments for humanity.

### 1.3. Design Philosophy of BIIH

We recognize that citizens’ choices of transportation mode largely depend on the urban built environment and the availability of transportation facilities. Constructing a strong sense of spatial belonging and psychological security for travelers can significantly influence their transportation decisions. Current research predominantly focuses on improving the cycling environment, while there is a deficiency in studies that fundamentally address the psychological well-being of cyclists. With the rapid advancement of modern technologies such as voice recognition and synthesis, as well as artificial intelligence-assisted communication technologies, it is now possible to provide more efficient, convenient, and safe transportation measures for urban cyclists. These technologies can help build a sense of cycling security, reduce psychological stress associated with urban travel, and enhance the overall cycling experience.

Existing fixed urban monitoring devices (e.g., traffic cameras, “cat’s eye” speedometers, and noise monitoring stations) can provide a macroscopic assessment of traffic conditions but fail to quantify the interaction between individual cyclists’ psychological states and their environment. They also cannot capture the fine-grained spatiotemporal characteristics of sudden stress-inducing events during cycling, such as unexpected parking or close overtaking. Traditional psychological survey methods, like questionnaires, have long-standing issues with subjective bias and incomplete data. Static sensors, on the other hand, have blind spots that reduce cyclists’ awareness of micro-stressors (e.g., sudden honking by motor vehicles) when traveling through these areas. Recent studies have explored various wearable technologies for cyclists. Elliot et al. developed a wearable undershirt to measure respiratory and heart rate parameters during cycling, but the study was limited to elite male cyclists and lacked comprehensive functional assessment [72]. Mejia et al. created a low-cost wearable system integrating sensors and LED lights to monitor environmental light and riding status in real time. However, the user test had a small sample size, lacked diversity in body types and riding habits, and provided insufficient feedback on comfort and wearability [73]. Boudreaux et al. evaluated the accuracy of several wearable devices during graded cycling and resistance training [74]. They found that most devices only measure heart rate at a single point in time and do not address measurement bias under physiological stress conditions such as dehydration and high temperatures, limiting their applicability.

The application of Internet of Things (IoT) sensors and affective computing-driven wearable devices offers innovative solutions to mitigate psychological load during urban travel. For instance, physiological sensors can monitor cyclists’ heart rate variability and galvanic skin response (GSR) in real time to identify physiological signals of stress and anxiety. Environmental sensors can synchronize the capture of surrounding traffic flow, natural environmental elements, and real-time user feedback to construct a dynamic situational stress model.

Based on current research, we are acutely aware of the urgent need for urban cyclists to perceive sudden stress-inducing events at the micro-scale of urban paths. Current studies suffer from insufficient urban data collection density, inadequate sensing systems for the psychological aspects of cycling, and high customization barriers for hardware. Moreover, there is a need for high response speed and multimodal data fusion capabilities, which require the integration of technical modules such as embedded system design, real-time signal reception and processing, and low-power communication protocols.

Our team, focusing on urban cycling, proposes a piece of intelligent interactive emotion-relief equipment. The helmet designed based on bionics can reduce its own weight while ensuring its rigidity and improving ventilation, which can alleviate the negative emotions compared with those of cyclists wearing traditional helmets. Meanwhile, due to the improved performance, it can ensure that the horizon and range of motion of cyclists are not affected by the shape and weight of the helmet, thus guaranteeing their ability to observe the environment of the street, and the ever-changing state of traffic safety. Starting from the perspective of interactive feedback mechanisms, this equipment provides emotional comfort to cyclists through environmental monitoring and interactive mechanisms. Its real-time interactive functions enhance the cycling experience, improve the wearer’s environmental perception, increase their sense of security, and alleviate anxiety. By doing so, we aim to gradually increase the proportion of cycling as a mode of transportation, promote healthy travel, and build and improve a green transportation system.

## 2. System Requirements and Design

The STM32 (STMicroelectronics, Geneva, Switzerland) is a 32-bit ARM Cortex-M microcontroller introduced by STMicroelectronics. It features high performance, low power consumption, a rich set of peripherals, and extensive applications, making it suitable for a wide range of application areas. The primary functions of the STM32 include providing a processor core, memory, and various peripherals, as well as supporting multiple development tools and software libraries to help developers quickly design and implement various applications [75]. It can achieve stable and precise control through built-in communication interfaces and high-speed timers and supports various low-power modes to reduce power consumption and extend battery life effectively.

### 2.1. Basis of the System Design

The microcontroller used in the BIIH is the STM32 F103C8T6 (Table 1). It is a cost-effective microcontroller with robust computing and control capabilities. This microcontroller employs an ARM Cortex-M3 core, with a maximum clock frequency of 72 MHz, and integrates a variety of peripheral modules, such as ADC, DAC, USART, SPI, and I2C, providing electronic engineers with a highly convenient development platform. The microcontroller features a modular circuit design and supports sensor hot-swapping, offering endless possibilities for developers. It is suitable for designers, non-professional programmers, and non-professional embedded system developers. Compared with the simple open platform Arduino, such as Arduino Uno, which is limited by its clock frequency of 16 MHz and memory resources of 2 kb SRAM, data delay issues may occur. When processing physiological signals in real time (e.g., GSR sampling rate > 100 Hz), data loss or delay (error rate > 12%) occurs, making it challenging to meet the low-latency requirements for affective interaction (ideal threshold < 200 ms). STM32 significantly outperforms Arduino boards in terms of real-time performance and processing speed, making it suitable for applications that require high performance, low power consumption, and powerful functionality. Due to its rich communication interfaces, and timer resources, it is also widely used in fields such as wireless communication, audio processing, and data storage.

This study integrates bionics principles in the design of form and structure to ensure safety and comfort. Additionally, the aerodynamic design reduces wind interference during high-speed cycling. The device, the BIIH, is a wearable interactive display device with human–machine interaction (HMI) capabilities that can interactively express corresponding elements by collecting users’ voice commands. The input signal integrates various electronic components, including temperature and humidity sensors, ultrasonic sensors, light sensors, voice modules, fans, LEDs, and OLED screens (Table 2). Integrating multimodal sensors and a real-time voice feedback mechanism provides real-time responses to the signal conditions of urban roads, forming an intelligent cycling helmet that can perceive the environment and interact with humans and the environment.

**Figure 1:**
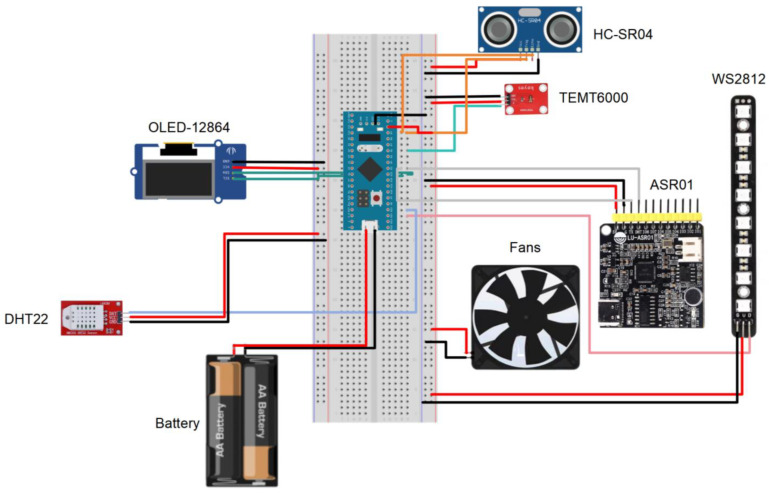
Electronic circuit of the BIIH. The BIIH comprises a variety of sensors including temperature and humidity, light, voice modules, ultrasonic sensors, and fan modules, which are connected to PWM ports. Electronic components such as LEDs, OLED screens, and other components connect the sensors to STM boards via expansion boards for multi-channel power supply and data reception. The English letters in the figure represent the model and type of different electronic components, which were selected based on the validation results presented in Section 3. The red wire indicates the live wire, the black wire indicates the ground wire, and the other colors are used to distinguish the signal wires for different components.

The STM32 F103C8T6 board has sufficient pins and computational power to accomplish this task. It features 37 digital General Pins of In and Out (GPIO) and 10 analog pins. The sensors used in the BIIH are based on the Transistor–Transistor Logic (TTL) protocol and will be connected to the digital pins. We have determined the response times of the sensors and SMDs (Figure 1). The size and weight of the STM32 F103C8T6 are highly suitable for wearable applications. With a weight of only 9 g, it can be comfortably and conveniently placed on a helmet without compromising comfort or causing the inconvenience associated with larger devices. The HMI device based on STM32 can achieve low-cost, delay-free interactions. The response time accuracy reaches the millisecond level, which can be effectively integrated with a well-designed motion capture system to achieve real-time HMI.

### 2.2. Choice of Hardware

The manufacturing budget for the BIIH is composed of multiple components, with the total budget for the base module components being approximately USD 46.4 (Table 3). The cost of the expansion components varies with the market price of the components, but the total cost will not exceed USD 80.

### 2.3. Open-Source Programming

**Figure 2:**
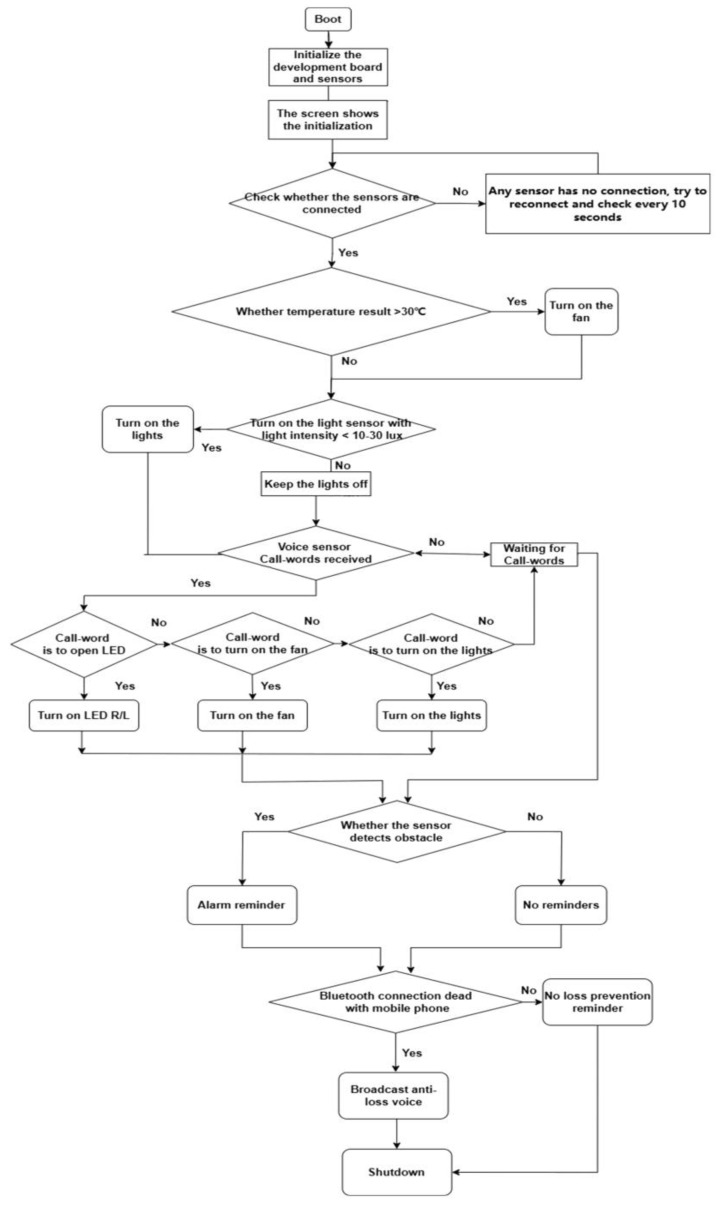
Flow Chart of BIIH.

The hardware programming of the device utilizes STM32CubeIDE, the official cross-platform IDE from ST, which is freely available and open-source under the GNU General Public License (GPL) and supports Linux, Windows, and macOS operating systems. The development of the device is centered around the C and C++ languages, achieving efficient embedded development through the STM32 Hardware Abstraction Layer (HAL) library and low-level register operations. The program flow is as follows (Figure 2).

### 2.4. Biomimetic Design

Biomimetic design is a method that imitates the structure, function, and behavior of biological organisms to solve engineering and technical problems [76]. By emulating biological entities in nature, biomimetic design offers novel approaches and solutions to engineering and technical challenges. It not only enhances the performance and efficiency of products but also possesses innovation, sustainability, and aesthetic value [77]. Through interdisciplinary collaboration and application across various fields, biomimetic design is driving technological progress and improving the quality of life.

To enhance the comfort of helmet wear, we attempted biomimetic design by emulating mature solutions in nature and the protective mechanisms of biological organisms to create safer products. This approach reduces the helmet’s weight while ensuring or even enhancing its structural rigidity. By mimicking the energy utilization methods exhibited by biological organisms over long-term natural selection and evolution, we reduced wind resistance to achieve energy savings. Additionally, we designed products with more naturally aesthetic forms.

#### 2.4.1. Morphological Design Based on Bionics

**Figure 3:**
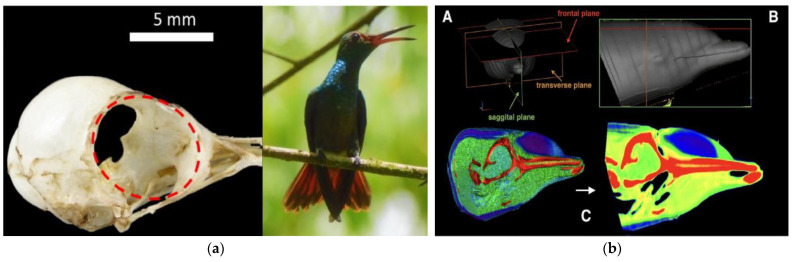
Morphological design based on bionics can reduce aerodynamic drag, optimize dynamic performance, decrease energy loss, and enhance structural integrity. (**a**). *Amazilia tzacatl* and its head bone structure, the red dot circle shows the dimensions of the hollow parts in the hummingbird’s skull; (**b**) dolphin’s head bone structure. A is the introduction of planes, B is the side view, C is the mechanical structure distribution on saggital plane.

To achieve the primary goal of ensuring helmet energy efficiency and reducing wind resistance, we conducted a comprehensive study and comparison of various biological forms, with a particular focus on the head shapes of birds and dolphins. The streamlined head shape of birds allows for efficient air penetration during flight. Integrating these morphological characteristics into helmet design can significantly reduce the drag coefficient during cycling, thereby decreasing the energy expenditure required to overcome wind resistance. Additionally, the skeletal structure of birds’ heads is lightweight yet rigid, ensuring the helmet’s protective performance. Similarly, the head of a dolphin, with its unique streamlined shape that has evolved over time, greatly reduces resistance while swimming (Figure 3). Applying this shape to helmet design can effectively optimize the helmet’s aerodynamic performance, reducing energy loss during cycling. Moreover, the construction of a dolphin’s head demonstrates excellent stability when withstanding water impact, which helps maintain the helmet’s structural integrity upon accidental impact. Overall, incorporating the head shapes of birds and dolphins into helmet design leverages their respective advantages in reducing wind resistance and energy consumption, providing cyclists with more efficient and safer protective gear.

#### 2.4.2. Rigidly Guaranteed Internal Structure of Circulation

The internal structure design of the helmet is crucial for impact protection and air circulation, directly affecting the wearer’s comfort. Therefore, we compared natural structures such as honeycomb, the internal tissue of a pomelo, and trabecular bone.

**Figure 4:**
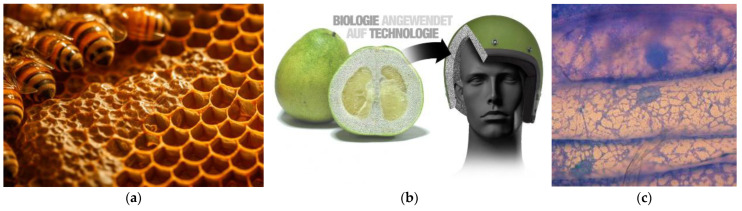
Comparison of a honeycomb structure, the internal tissue of a pomelo, and trabecular bone structure. (**a**) Honeycomb possessing characteristics of high strength and low weight; (**b**) BMW’s new pomelo-inspired helmets have up to 20 percent better protective properties; (**c**) morphology of the cytoskeleton under a microscope, keeping the internal structure of the cell in order.

The honeycomb structure, with its regular hexagonal cell arrangement and offers good compressive strength while being lightweight and effectively dispersing impact forces, which is significant for the helmet’s impact resistance. The internal tissue of pomelo has a complex yet orderly structure, with gaps between its lobes providing natural air channels that enhance air circulation inside the helmet, reducing stuffiness and improving the wearer’s comfort. Trabecular bone, a naturally porous structure found in animal skeletons, is known for its excellent mechanical properties and lightweight nature. We employed a trabecular bone algorithm for the helmet’s internal structure design [78] (Figure 4). This algorithm generates trabecular-like structures that precisely distribute material, ensuring sufficient impact resistance while minimizing weight. The pores in the trabecular structure also facilitate air circulation, significantly enhancing comfort and achieving a good balance between impact protection and ventilation. To evaluate the structural rigidity, we used professional software like Flow 1.0 to simulate the performance of these designs in cycling scenarios, quantitatively analyzing various performance indicators to select the optimal helmet design, ensuring its scientific basis and reliability.

To validate the actual performance of the biomimetic BIIH prototype, we conducted a series of rigorous experiments. These experiments focused on testing structural rigidity by simulating various extreme loading scenarios to determine if the helmet could provide reliable protection under different conditions. We also tested wind resistance and airflow, using software simulations to precisely measure the wind resistance at different speeds and the airflow over the helmet’s surface. This assisted us in continuously optimizing the design to ensure the helmet’s safety and comfort in practical application.

In the cutting-edge field of design, biomimetic design has demonstrated exceptional innovation [79]. It can seamlessly integrate sensor modules with the helmet’s form, using ingenious construction and layout to minimize the awkwardness of module installation. For example, by mimicking the natural contours and structural characteristics of biological heads, the sensor modules appear as if they naturally grow on the helmet, perfectly matching the overall design. The incorporated natural aesthetic elements, from soft lines to natural color schemes, effectively reduce the psychological stress of cyclists during rides. This biomimetic design not only significantly enhances the helmet’s functionality, allowing sensors to operate more accurately and stably but also creates a more harmonious human–computer interaction experience. Cyclists can interact with the helmet more naturally, providing a more nature-oriented solution for emotional assessment and stress relief, breaking away from the limitations of traditional design.

## 3. Functional Validations

The BIIH designed in this study aims to provide emotional comfort for the cycling community on urban roads, enabling cyclists to perceive the surrounding space better and enhance their sense of security.

### 3.1. Experimental Design

To ensure the consistency, integrity, and scientific nature of this device in terms of structure and function, we designed a series of experiments.

Firstly, we verified whether the BIIH helmet could enhance the wearer’s environmental perception ability, including the monitoring of temperature, humidity, light intensity, and the approach of objects. We selected control group sensors by model or brand for these sensors and used a microcontroller to collect real-time data on temperature, humidity, light intensity, and distance. Through a data analysis, we evaluated the accuracy of different sensors in understanding environmental information.

Secondly, we tested the performance of the BIIH helmet in improving specific stiffness based on bionic design verification. We used professional mechanical structure testing software and airflow verification software to conduct simulation verification on the structural rigidity and ventilation performance of the bionic helmet.

Finally, we detected and verified the real-time interaction function of the BIIH helmet. In an experimental site simulating an urban traffic environment, we set up various types of background noise to verify whether the interaction system could accurately and quickly capture valid information and achieve interaction.

We fully considered all possible factors that could affect the experimental results. By means of reasonable experimental grouping and comparative experiments, we minimized experimental errors.

### 3.2. Thermos-Hygrometer Sensor

The BIIH aims to alleviate the psychological stress of wearers and enhance their comfort during cycling. It is equipped with a thermos-hygrometer sensor. By continuously monitoring the temperature and humidity conditions around the wearer’s head, the device can adjust the state and speed of the built-in fan, ensuring that the wearer always enjoys the most comfortable wearing environment.

This study compares the typical temperature and humidity sensors. The most widely used sensors are DHT11 and DHT22. Luo et al. have previously conducted comparative experiments on these two sensors and showed that DHT22 exhibited more precise sensing capabilities and more stable environmental sensing performance than DHT11 [78]. The purpose of using a temperature and humidity sensor in this project is to monitor the temperature and humidity conditions around the human head in real time. Therefore, based on the work of our previous research, we innovated the experiment by comparing DHT11 and DHT22 again and adding the temperature sensor AS6221, which is commonly used in commercial forehead thermometers (Table 4). An industrial-grade commercial digital temperature and humidity meter equipped with TA622C was used for synchronous monitoring to serve as a control.

**Figure 5:**
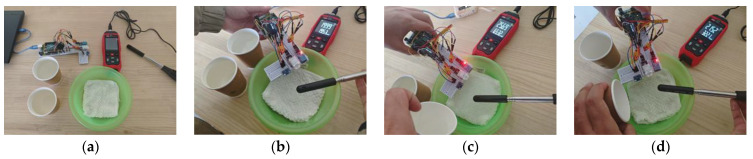
Comparison of temperature and humidity sensors. (**a**) Preparation and arrangement of instruments and equipment. (**b**) The three sensors and the control group device were placed at the same height and kept stationary for real-time monitoring. (**c**) Pouring a small amount of hot water onto the towel to stimulate the response of the temperature and humidity sensors. (**d**) Pouring a small amount of cold water onto the towel to stimulate the response of the temperature and humidity sensors.

To effectively test the accuracy and sensitivity of the temperature and humidity sensors under normal operating conditions of the BIIH, the experiment was conducted in a standardized indoor laboratory. The experiment aimed to simulate the sweating state of a user under the helmet, and the process was determined to involve soaking a towel in warm water and wringing it out to a slightly damp state to mimic the condition of the human head about to sweat. The three sensors were suspended above the towel for real-time monitoring to simulate the sweating state under the helmet. Four different ambient temperatures were used to stimulate and compare the response sensitivity and accuracy. As shown in Figure 5, during the experiment, hot and cold water were repeatedly poured onto the towel to simulate different sweating environments of the human head and to compare the response sensitivity and accuracy of the three sensors.

**Figure 6:**
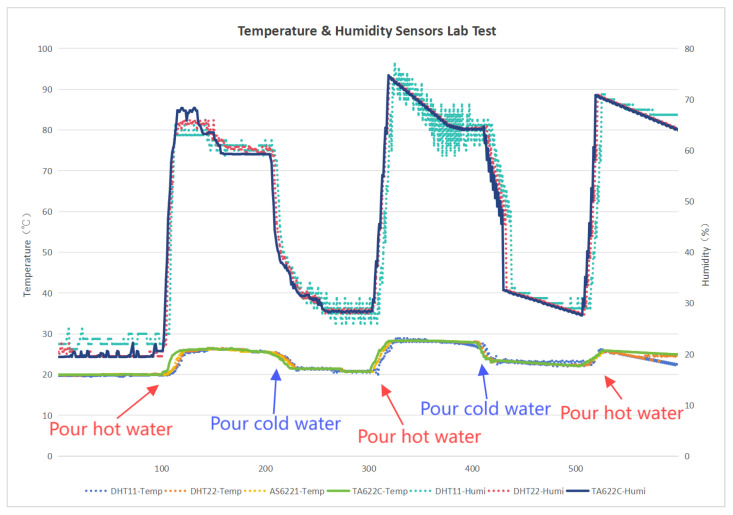
The comparison of 600 data points from DHT11, DHT22, AS6221, and TA622C under different modes.

We collected 600 sets of data to compare the temperature and humidity conditions of DHT11, DHT22, AS6221, and TA622C under the same time and scenario. In the temperature-sensing performance test, DHT11 had a percentage difference of less than 2% in 75.68% of cases, while DHT22 had a percentage difference of less than 2% in 98.78% of cases and less than 0.5% in 68.78% of cases. AS6221 had a percentage difference of less than 0.5% in 79.89% of cases. In the humidity-sensing performance test, DHT11 had a percentage difference of less than 2% in 58.33% of cases, while DHT22 had a percentage difference of less than 2% in 76.18% of cases (Figure 6). In terms of temperature and humidity sensing, DHT22 outperformed DHT11 in both sensitivity and accuracy. AS6221 slightly surpassed DHT22 in sensitivity and accuracy for temperature sensing. Through comprehensive comparisons in a laboratory environment, we determined ASDHT22 as the final choice for the temperature and humidity sensor.

### 3.3. Light-Intensive Sensors

During urban cycling, the lighting environment, as an essential factor in ensuring cycling safety, plays a crucial role in the physiological safety and mental health of cyclists [80]. For the evaluation coefficients of shadow and lighting environments in urban cycling, data are primarily collected using data acquisition vehicles. When recording ambient light during experiments, the BIIH provides data support for the multidimensional impact factor research on cycling comfort experience by integrating GPS module data and temperature and humidity sensor data, as well as by monitoring changes in surrounding environmental illumination values during the cycling route [72,81].

The venues for the environmental lighting experiments were divided into two scenarios: an outdoor environment with sufficient lighting conditions and a tunnel environment with obstructed lighting conditions. In this comparative experiment, considering practical application scenarios, the experimental subjects were ultimately determined to be three types of light sensors: the miniature light sensor TEMT6000, BH1750, and the Microbit light-sensitive module. The data collected in this experiment will be compared for further selection (Table 5).

In accordance with the experimental design requirements, the TEMT6000, BH1750, and Microbit light-sensitive modules were placed in environments with the same illumination levels. The three sensors were positioned 10 cm away from the light source, which was operated in both the off and on modes, to test the sensitivity of data recording and the reliability of instrument operation under actual cycling conditions. During the experiment, the quality of the light source should be noted to ensure that the light cone has a sufficient diameter to provide uniform light signals to the different sensors.

**Figure 7:**
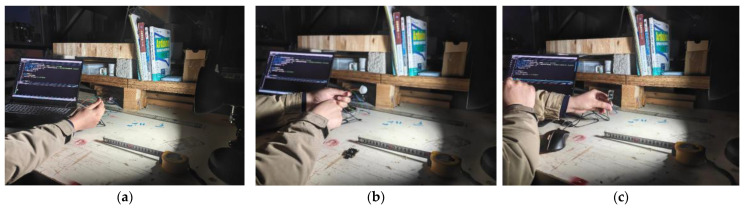
Comparation of light-sensitive modules. (**a**) Light-intense test with TEMT6000; (**b**) light-insensitive test with BH1750; (**c**) light-insensitive test with the Microbit light-sensitive module.

To ensure the authenticity and reliability of the experimental data, the real-time measurements for all three sensors were monitored using the serial port monitor. The experimental duration was set to 20 s. Based on the actual test results, after analyzing the data fluctuations recorded by the serial port monitor within the same time frame, it was found that the BH1750 had a slower response (Figure 7). To ensure the stability and reliability of the experimental data, the BH1750 was excluded.

To determine the potential of the sensors for practical applications, we chose to compare the practical application data of the TEMT6000 and the Microbit light-sensitive module.

**Figure 8:**
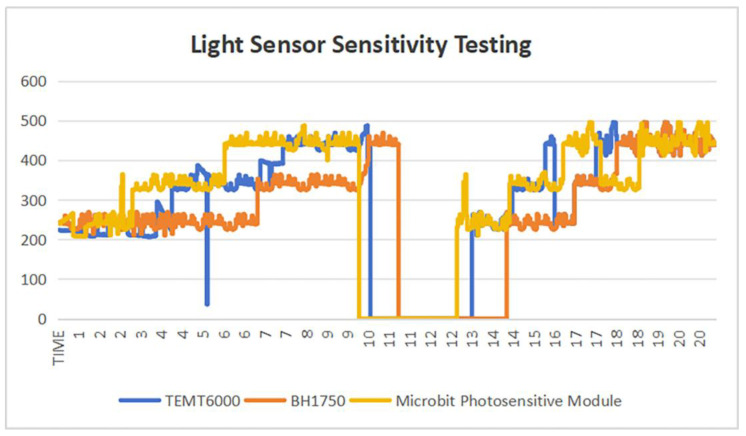
The test data on the sensitivity of TEMT6000, BH1750, and the Microbit light-sensitive module to light sources were measured.

In the real-world usage comparison adopting TEMT6000, BH1750, and the Microbit light-sensitive module, the sensor module for future BIIH applications is required to be small in size and relatively low in power to prevent hazards to cycling safety. During actual cycling, the response time of the light-sensing module must be rapid and stable. The response time advantage of TEMT6000 over the Microbit light-sensitive module is very evident. Considering the purchase cost, the cost of the Microbit light-sensitive module is more than ten times that of the TEMT6000 module, which does not align with the design philosophy of BIIH (Figure 8). Ultimately, TEMT6000 was selected for further research. Although the Microbit light-sensitive module was found to be more precise in experimental data recording during practical applications, the significant cost difference led to the final choice of TEMT6000.

### 3.4. Distance-Measuring Sensor

The data from the ultrasonic sensor primarily aimed at addressing safety issues during urban cycling, especially when multiple vehicles are riding abreast. The main objects of concern in cycling are cyclists and motor vehicles. The ultrasonic sensor was combined with LED and voice modules to provide sufficient safety measures for urban cycling. The HC-SR04 ultrasonic sensor was selected as the experimental module for this study. This sensor used the principle of ultrasonic pulse emission to measure objects within the sensing range accurately. Given its high-frequency ultrasonic emission, the data error is ±0.2 cm, and the emission frequency is 40 kHz (Table 6).

**Figure 9:**
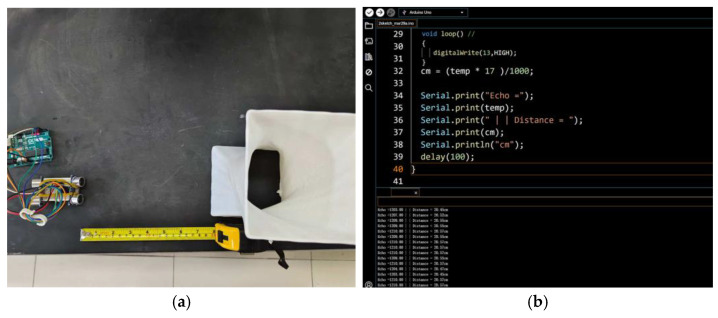
The actual distance measured by ultrasonic ranging. (**a**) Experiments utilize HC-SR04; (**b**) data from the ultrasonic sensor read via the PCB serial port.

The BIIH is equipped with two sets of HC-SR04 ultrasonic sensors to collect environmental data on the left and right sides of the cyclist during cycling. We programmed the ultrasonic sensors using the Arduino IDE for serial communication via serial code. During this study, data were recorded using the Arduino Serial Monitor. The validation experiment for the ultrasonic sensors was conducted in a laboratory setting. The experiment confirmed the data acquisition capability of the HC-SR04 ultrasonic sensors by comparing the collected data with actual distances. Data were read using the Serial Monitor in the Arduino IDE, and through code programming, the HC-SR04 can record 100 to 200 data sets within a few seconds (Figure 9).

**Figure 10:**
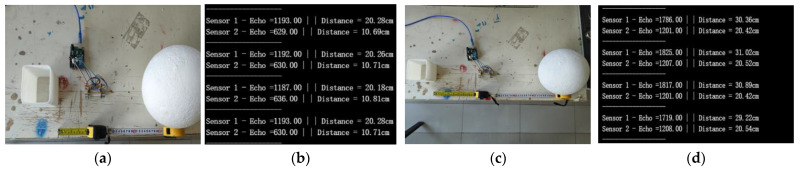
The actual distance measured by ultrasonic ranging. (**a**) Group A experiments; (**b**) Group A experiment results read via the Arduino IDE; (**c**) Group B experiments; (**d**) Group B experiment results read via the Arduino IDE.

Upon testing, we obtained accurate data, which validated the feasibility of incorporating the HC-SR04 ultrasonic sensor module into the BIIH. During actual cycling, the BIIH requires both left and right ultrasonic sensors to operate in tandem to guard against potential hazards. We improved the original single-side ultrasonic sensor module to enable it to detect and capture the distance of objects on both sides of the module. Subsequently, we conducted multiple data collections using the HC-SR04 ultrasonic sensor. After comparing several sets of serial data, we utilized the STM board’s serial ports for data acquisition. In the laboratory, we verified the accuracy of the HC-SR04 ultrasonic sensor by comparing different reference objects and distances (Figure 10).

In the Group A experiment, the actual distance from the HC-SR04 sensor module to the cube on the left side was 10 cm and to the sphere on the right side was 20 cm. In the Group C experiment, the actual distance from the HC-SR04 sensor module to the cube on the left side was 20 cm and to the sphere on the right side was 30 cm. The comparison of the actual data and the data read via the Arduino IDE Serial Monitor showed an error of ±0.5 cm, which was within the acceptable range. The HC-SR04 sensor module generally meets the application requirements and is in line with the requirements of the BIIH.

### 3.5. Validation of Bionic Structures

Introducing bionic concepts into helmet design aims to create products that are safer, are energy-efficient, and provide a comfortable experience. To ensure that the helmet structure and its wind resistance function effectively meet the intended purposes, we conducted comprehensive functional validation.

#### 3.5.1. Structure Validation of BIIH

This study used the Rhinoceros (version 7.3) software to construct the bionic design model of the helmet precisely, and it combined its plugin Ameba to conduct structural simulation experiments on the helmet. The stress–strain distribution of the helmet structure was analyzed to identify weak points and the distribution of rigidity. Experimental data from different bionic helmet designs and traditional helmets were compared to assess the advantages and limitations of bionic structures in enhancing rigidity.

(1) Model Establishment

**Figure 11:**
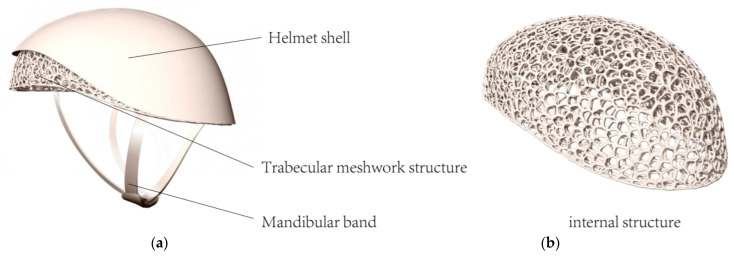
BIIH bionic designed structure. (**a**) The shell design mimicked a hummingbird’s head; (**b**) the BIIH inside form design was generated with the trabecular algorithm.

The helmet’s shape mainly includes the outer shell, inner mesh structure, and chin strap, with comfort padding ignored for its minor impact on simulation (Figure 11). The outer shell was modeled based on the bionic morphology of a hummingbird skull, and the inner structure was modeled using the trabecular algorithm. The trabecular structure mesh lining of the BIIH is the primary component that provides structural rigidity. It absorbs impact energy through permanent compressive deformation, thereby reducing the risk of head injury. In the mechanical structure validation of the helmet, this mesh structure bears significant impact loads.

(2) Setting Simulation Parameters

**Figure 12:**
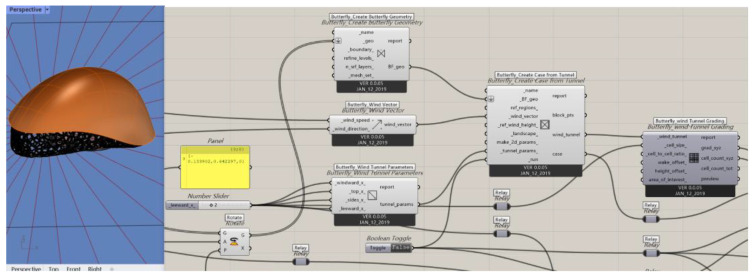
The mechanical structure of BIIH is configured with Ameba.

The Ameba plugin was used to configure the mechanical structure validation of the helmet. The material properties of the helmet were defined. For carbon fiber material, the elastic modulus was set at 230 GPa, the Poisson’s ratio at 0.3, and the density at 1.75 g/cm^3^. Constraints were set to simulate the helmet’s wearing state, with fixed constraints applied to the areas in contact with the head. Loads were added to account for potential impacts, with a concentrated force of 500 N applied vertically downward at the top of the helmet (Figure 12).

(3) Simulation Results

After connecting all components in Grasshopper and ensuring correct parameter settings, the mechanical structure validation using Ameba was run. During the simulation, attention was paid to the progress of the calculations and any error messages that might appear.

**Figure 13:**
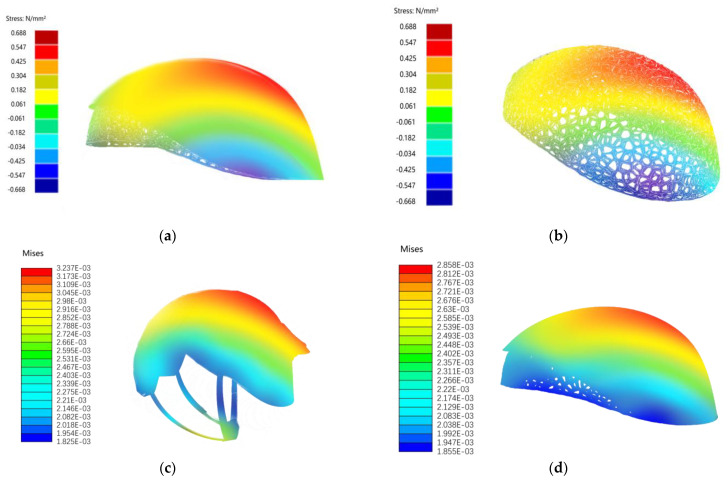
The results of the structural analysis experiments. (**a**) Mechanical structure stress distribution of the BIIH helmet; (**b**) mechanical structure stress distribution of the BIIH inside its form. (**c**) Stress cloud map of traditional helmets; (**d**) stress cloud map of BIIH.

Through structural analysis experiments, we found that when a concentrated force of 500 N was applied to the top of the helmet, the stress distribution map showed stress concentrations at the edges in contact with the fixed constraints and some key connection points, with the maximum stress reaching 100 MPa. The maximum strain of the helmet appeared in the loaded area at the top of the helmet, at approximately 0.001 mm/mm (Figure 13). An analysis revealed that these areas need to be reinforced during actual use to enhance the helmet’s impact resistance.

In terms of structural rigidity validation, the stress range of the conventional helmet is from 1.825 × 10^−3^ to 3.237 × 10^−3^, while that of the BIIH (Biomimetic Integrated Impact Helmet) is from 1.855 × 10^−3^ to 2.858 × 10^−3^. The relatively lower upper limit of stress values in the BIIH indicates that, under identical loading conditions, the maximum stress experienced by the BIIH is lower than that of the conventional helmet, thereby conferring a certain advantage in resisting high-stress damage. As observed from the color distribution in the stress maps, the high-stress regions (indicated in red) of the BIIH are concentrated at the top, suggesting that this area is the primary load-bearing region. In contrast, the low-stress regions (indicated in blue) are located in the lower areas, implying that these regions experience lower forces. This relatively localized stress distribution facilitates targeted reinforcement of the top structure, thereby enhancing its protective performance. In contrast, the high-stress regions of the conventional helmet are not only present at the top but also distributed across other areas, resulting in a more dispersed stress distribution. This indicates that the force transmission and distribution in the conventional helmet are more complex, lacking a distinct primary load-bearing region. Therefore, structural optimization of the conventional helmet requires a comprehensive consideration of multiple areas, making the optimization process more challenging [82].

The BIIH can focus on enhancing the strength and cushioning performance of the top structure to further reduce the stress concentration at the top during impact. In contrast, the conventional helmet requires a comprehensive evaluation of the structures at all locations to identify the sources of stress concentration and optimize the overall structural design to improve the stress distribution. In terms of protective performance, the BIIH helmet, with its stress concentrated at the top, can effectively disperse and absorb energy through rational top design, potentially offering more targeted protection for critical head areas. However, the conventional helmet, with its dispersed and complex stress distribution, may fail to provide adequate protection for certain areas if not properly designed. This effectively dispersed the impact force and protected the internal simulated head model from severe damage.

#### 3.5.2. Morphological Drag Validation of BIIH

Regarding the drag of bionic shapes in motion, we utilized the Ladybug 1.8.0 plugin in Grasshopper to assess the performance of the helmet structure in practical applications. This involved simulating and measuring the drag coefficient of the helmet under various cycling conditions to explore its aerodynamic characteristics. By doing so, we aimed to determine whether the energy-saving and ventilation effects met expectations, providing data to optimize the design, enhance cycling comfort, and ensure the safety of the cyclist’s head.

Based on the completed model, the helmet was imported into Grasshopper for subsequent simulation and analysis.

(1) Wind Environment Simulation

**Figure 14:**
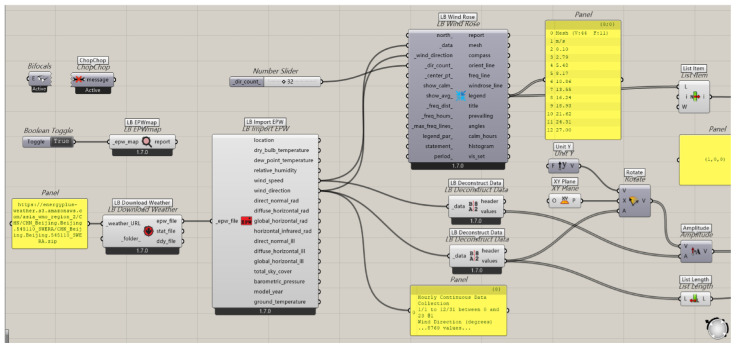
Parameter setting for wind environment in Ladybug.

The Ladybug plugin was used to set the parameters for wind environment simulation (Figure 14). The wind direction was determined based on common cycling scenarios, with the wind direction set horizontally at an angle of 0°. The wind speed was set at 10 m/s (equivalent to 32 km/h), referencing the cycling speed range in Beijing. The atmospheric boundary layer height was set at 300 m, assuming an urban street environment. The ground roughness was selected based on different road surface conditions (e.g., asphalt and concrete), with a value of 0.1 m chosen.

The visualization tools of Ladybug were used to observe the flow of air over the helmet surface, identify any turbulence or vortex regions, and obtain streamline and pressure distribution visualizations of the airflow around the helmet. Pressure values at different locations on the helmet surface were read to analyze the pressure distribution pattern.

(2) Simulation Results

**Figure 15:**
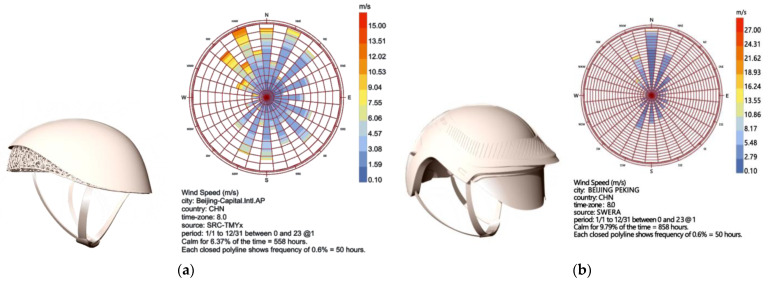
The results of the wind environment simulation. (**a**) BIIH wind environment verification diagram; (**b**) ordinary helmet wind environment verification diagram.

At a wind speed of 10 m/s, the maximum pressure on the helmet surface appeared at the center of the front of the helmet at approximately 20 Pa; the minimum pressure appeared at the back of the helmet at approximately −5 Pa. The airflow streamline diagram showed that the airflow over the top and sides of the helmet was relatively smooth, but a small amount of turbulence appeared at the back of the helmet, indicating that the design of the back of the helmet may need further optimization to reduce turbulence (Figure 15).

Based on the data results, within the same wind speed range (0.5–10.5 m/s), the lift coefficient range of the bionic helmet was 0.10–15.70, while that of the conventional helmet was 0.10–27.60. The lower upper limit of the lift coefficient for the bionic helmet means that it experiences relatively less upward lift in the wind environment, making it less likely to shift or wobble upward when worn, thus providing better stability. The drag coefficient range for the bionic helmet was 0.50–15.00, compared to 0.50–20.00 for the conventional helmet. The lower upper limit of the drag coefficient for the bionic helmet indicates that it experiences less wind resistance during activities such as cycling, reducing energy loss and improving efficiency. In terms of the moment coefficient, the range for the bionic helmet was 0.00–3.10, while for the conventional helmet, it was 0.00–9.60 (Table 7). The smaller moment coefficient suggests that the bionic helmet has a weaker tendency to rotate around its axis under wind forces, better maintaining the stability of its wearing orientation and reducing the interference of wind on the helmet’s posture.

The results show that in the drag validation, the bionic helmet has a significantly lower drag coefficient than traditional helmets. It maintains a low level of wind resistance under different wind speeds and directions. The helmet can guide airflow more smoothly over the surface, reducing turbulence and vortex formation. This enhances aerodynamic comfort during cycling, achieves significant energy-saving effects, enables cyclists to travel longer distances with the same energy expenditure, and reduces fatigue caused by airflow interference.

Through the comprehensive and rigorous validation process of the bionic structure, we gained an in-depth understanding of the performance of bionic design helmets in practical applications. This provides a solid and reliable scientific basis for further optimizing the design and improving product functionality, promoting the widespread application and innovative development of bionics in helmet design.

### 3.6. Verification of Voice Interaction Modules

**Figure 16:**
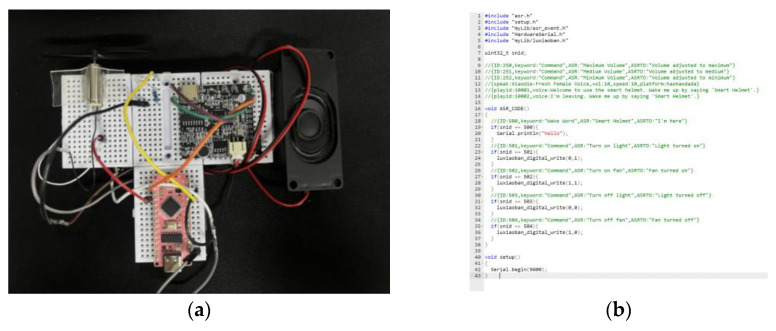
Functional verification of the voice module. (**a**) The voice module wired with STM32; (**b**) experiment results of the voice module.

To ensure the reliability of the human–machine interaction functions of the smart cycling helmet, this study conducted functional verification of the voice module. We assessed and verified the voice accuracy and anti-interference capabilities. The ASR01 module selected in this study can support 10–20 custom offline call-words, which meets the actual needs. For the efficiency of the experiment, as shown in Figure 16, a test platform was built using the STM32, and two functions, voice-controlled lighting and fan, were selected for verification.

Urban traffic conditions are complex and variable, with traffic noise accounting for more than 60% of the urban acoustic environment [83]. The primary source of environmental noise is the noise emitted by vehicles, which has a much more severe impact on cyclists. We conducted on-site measurements of multiple urban roads in Beijing, China, using a TA652B decibel meter. The noise sample collection covered different time periods (morning and evening rush hours, and off-peak hours) and various scenarios (main roads and side roads) to ensure the representativeness of the data.

**Figure 17:**
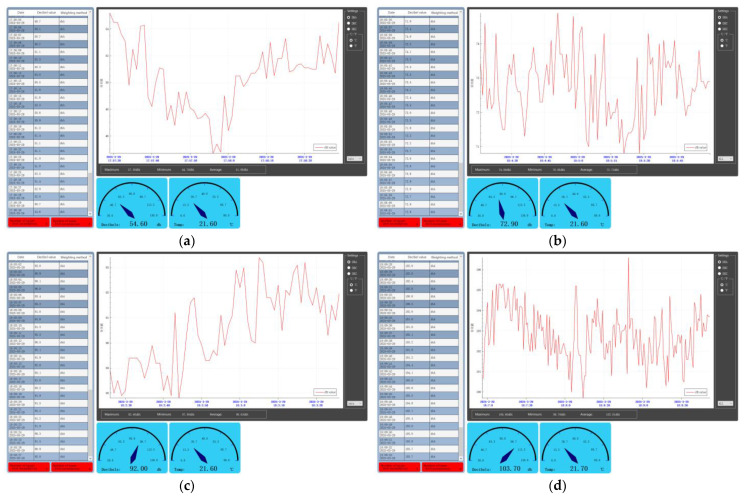
Urban traffic noise sampling. (**a**) Around 50 dB; (**b**) around 70 dB; (**c**) around 90 dB; (**d**) around 110 dB.

The voice verification module used must be able to function appropriately under different noise environments. We recorded a segment of white noise in the center of an urban road, as shown in Figure 17. By adjusting the loudspeaker volume, we simulated noise environments of 50 dB, 70 dB, 90 dB, 100 dB, and 110 dB.

In each noise environment, we executed 50 experiments for each of the call-words—**①** Turn on light; **②** Turn off light; **③** Turn on the fan; and **④** Turn off the fan—totaling 200 experiments per group. The experimental procedure is as follows: set the loudspeaker volume to medium, with the microphone in the ASR01 module positioned 30 (±5) cm from the sound source. The experiments were then conducted sequentially in each noise environment, with the results recorded based on feedback.

The experimental results indicate that the ASR01 module functions normally in noise environments of ≤90 dB. However, its performance significantly deteriorates in noise environments above 100 dB. This is mainly attributed to the voice signal being overwhelmed by noise and the variation in cyclists’ pronunciation in high-noise environments. Cyclists need to actively increase the volume of their commands to ensure the sensor functions properly.

- Low to mid-high noise environments (≤90 dB): The recognition rate of the ASR01 module reaches 100%, meeting practical requirements.
- High noise environment (100 dB): The recognition rate drops to 95.5% (191/200), and users need to increase the volume to above 85 dB to compensate for noise interference.
- Extreme noise environment (110 dB): The recognition rate plummets to 27.5% (63/200). The main reasons include the voice signal being overwhelmed by noise (signal-to-noise ratio < 5 dB) and user pronunciation variation, such as intonation distortion.

**Figure 18:**
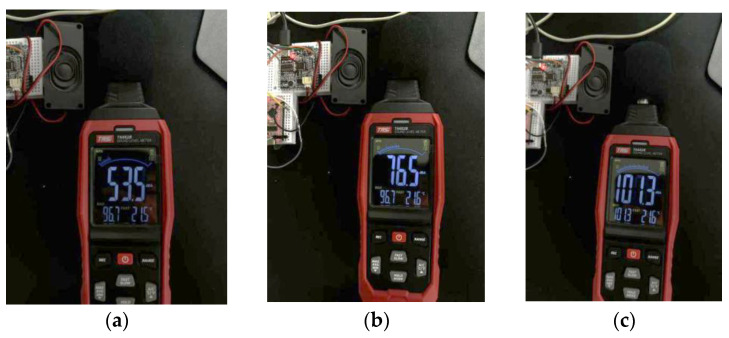
Speakers’ tests with three different volume levels. (**a**) Minimum volume used in a low-noise environment; (**b**) medium volume used in common traffic noise environments; (**c**) maximum volume used in high-noise environments.

Finally, we tested the loudspeakers of the sensor, which were configured with three different volume levels: minimum, medium, and maximum. Figure 18 shows the tests conducted on these three volume levels:

- In a low-noise indoor environment (<60 dB), the minimum volume mode was used.
- In a typical urban traffic noise environment (60–80 dB), the medium volume mode was used.
- In a high-noise environment (>80 dB), the maximum volume mode was used. Cyclists can adjust the volume according to the different environments.

## 4. Field Measurement Results

Ultimately, we invited 15 participants with different experiences in cycling to wear the BIIH and simulate a 20 km cycling trip in a typical urban setting (Appendix A). The continuous and real-time data collection revealed that the BIIH can efficiently and accurately gather data. It can also sensitively and accurately interact with pedestrians in the environment. By actively intervening, the BIIH can break the vicious cycle of stressors causing anxiety and avoidance behaviors. This helps wearers enhance their environmental perception and improve their sense of security during travel. The system validated the practicality of a cycling helmet with integrated multimodal sensors in a dynamic urban traffic environment. It provides a new paradigm for the application of intelligent wearable devices in specific scenarios.

### 4.1. Input and Output Settings

Based on the observation of cyclists’ overall travel behavior, we believe that enhancing people’s environmental perception and group interaction abilities through sensing devices can significantly increase their travel safety. Therefore, we set the scenarios for the BIIH to acquire surrounding environmental information as follows:

- When people need to make a left or right turn, the helmet can light up the turn signal through a voice command.
- When the helmet temperature exceeds the set temperature or humidity threshold, the cooling system will be activated directly. Alternatively, if the cyclist feels that the helmet temperature is too high to bear, they can command the cooling system through a voice command.
- When the surrounding environmental brightness is insufficient, the lighting system can be activated directly if the light sensor detects a value below the threshold. Alternatively, the cyclist can turn on the lighting system through a voice command.
- When there is a potential danger approaching outside the cyclist’s field of vision, the sensors can detect the distance and issue a sound alert.

### 4.2. Input and Output Results

We collected the following data through sensor integration, processed and fed back the data, and verified the BIIH’s sensitive response through user experiments. The voice interaction sensor embedded in the bionic helmet has a relatively high ability to recognize speech in noisy environments. We recorded the cycling process of each participant using a Pilot One 8 K panoramic camera (Labpano Technolog Co., Ltd., Changzhou, China) and collected environmental data through the video acquisition system. The obstacle detection accuracy rate was 85.4% (false alarm rate < 0.5 times per kilometer), and the average obstacle warning response time was 0.42 s, which is 60% faster than the average human visual reaction time [83]. The sensor’s rapid response and recovery time are both less than the speed recognizable by the naked eye (set by us in the actual wearing experiment as waiting for 0.5 s to respond after recognizing a voice command). The light regulation error is <5% (set by us in the actual wearing experiment as waiting for 0.5 s to respond after recognizing a voice command). In addition, the temperature and humidity sensor integrated into the BIIH showed high accuracy in cross-monitoring physiological signals, highlighting the device’s ability to effectively sense and respond to environmental and physiological stimuli, with a fan response time triggered by the temperature and humidity sensor of <1.5 s.

In this study, the voice interaction module remains an open-ended module. In the future, we plan to empower it to achieve information acquisition and sharing based on the cloud-edge-end architecture of a smart city through network connections. With the sharing of network information, cyclists, just like motor vehicle owners, can know changes in traffic lights one minute in advance and then adjust their cycling speed accordingly. They can also, based on whether there is congestion in the real-time cycling street space, combine with the real-time map navigation switching plan, such as passing through parallel alleys to shorten the journey. After being connected to the network and a dedicated cycling map, this module will play a greater role in the future and expand the application scenarios of the BIIH.

The above experiments demonstrate that the BIIH can accurately, efficiently, and intelligently intervene in the wearer’s traffic experience through light, temperature and humidity, obstacle detection, and multimodal feedback (voice prompts, and fan and light regulation), alleviating negative emotions.

**Figure 19:**
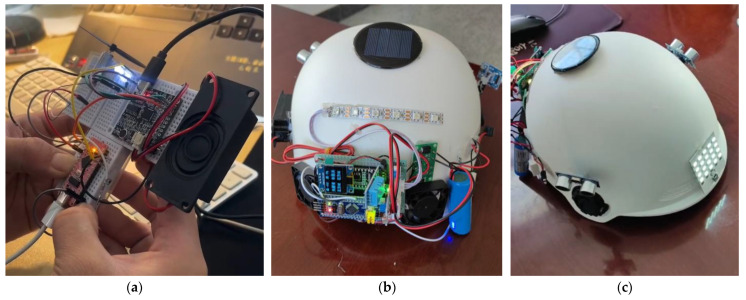
Interactive functional testing of the electronic component collection of the BIIH prototype: (**a**) the “Turn on light” call-words being expressed; (**b**) an electronically integrated rear view of the BIIH; (**c**) a side view of the BIIH’s electronic integration.

The device, powered by a portable power bank, can operate continuously for 1.2 h, meeting the duration requirements for daily cycling. During this period, the power bank provides sufficient energy to support the BIIH’s interactive functions, including voice interaction, lighting, and LED display (Figure 19). The device demonstrated stable performance overall. These results confirm that the device is suitable for wearable applications, ensuring portability and long-term wearability.

The integrated wearability of the BIIH is also crucial for cyclists. In real-life cycling scenarios, the anti-shake performance of bicycles and scooters mainly depends on their mechanical structures. Unlike cars, which can use suspension systems to reduce body vibrations, external devices such as mobile phones, navigation systems, and cycling computers for bicycles and scooters are difficult to install and remove. This often leads to damage or breakage of these devices due to vibrations. Additionally, when devices are placed on the handlebars, voice interaction becomes impossible during cycling. Cyclists cannot hear navigation instructions or use voice commands to request real-time information updates. As a result, many cyclists choose not to use any such devices, which is detrimental to improving their cycling experience.

We further reviewed the design philosophy and implementation strategies of the BIIH, with the expectation of achieving good application prospects in other wearable devices. The BIIH, with its modular design, bionic design concept, multimodal sensing components, and intelligent voice interaction system, successfully constructed a prototype for wearable traffic perception enhancement. This prototype demonstrated similar sensitivity and interactive capabilities, validating that the BIIH’s design approach can be extended to other wearable applications.

We designed a Likert scale to assess the usability and user-friendliness of the BIIH and collected feedback on user satisfaction (Table 8). Satisfaction was rated using a 5-point Likert scale, ranging from “Very dissatisfied” (scored as “1”) to “Very satisfied” (scored as “5”). According to the principles of the 5-point Likert scale, a perfect score is 5 points. A total score exceeding 3 indicates that the respondents are generally satisfied with the product’s performance, while a score exceeding 4 indicates a high level of satisfaction. As shown in Table 2, the respondents were highly satisfied with the performance of the product.

In summary, the results of this study indicate that the BIIH has high sensitivity, adaptability, and portability. These findings highlight wearable interactive devices as a new way of perceiving traffic environments and expressing intelligent decision-making. They also provide a foundation for the development of other intelligent wearable devices.

## 5. Discussion

In the context of the rapid development of modern cities, many urban dwellers face varying degrees of psychological distress. Situational anxiety among citizens in traffic scenarios is also on the rise, especially among cyclists who lack a sense of spatial belonging and security regarding this mode of transportation. To address this issue, we have designed and developed an interactive smart cycling helmet based on bionic principles. The BIIH is developed on the STM32-embedded development board and incorporates multimodal sensing components, including temperature and humidity sensors, ultrasonic sensors, light sensors, voice modules, fans, LEDs, OLED screens, and other electronic components, forming a smart cycling helmet capable of perceiving the environment, human–machine interactions, and environmental interactions. A portable power bank is used to ensure portability. The experimental results indicate that the BIIH has a highly sensitive signal response, reliable environmental perception, and more secure interactive capabilities. These characteristics collectively highlight its potential as a multifunctional and user-friendly wearable device.

Compared with traditional helmets, the BIIH has several significant advantages. The helmet is based on bionics for morphological design and structural validation. The bionic design validation of the helmet enhances specific stiffness. The device can provide real-time feedback based on sensor inputs, enhancing the wearer’s environmental perception and safety. It also enables real-time human–machine interactions, which helps improve the wearer’s traffic experience and alleviates the psychological stress of daily travel. Lastly, the device’s power and storage capabilities make it portable, independent, and iterative.

Despite the promising results, the current study still has some limitations that are worth further investigation. For example, the structural safety of the BIIH in sudden situations for users traveling at higher speeds, as well as its long-term durability and reliability when continuously used and exposed to environmental factors, need to be assessed through more extensive testing. During the validation of the bionic structure in this experiment, other commercial materials used in safety helmets should have been employed for empirical validation. However, due to equipment limitations, only PLA and carbon fiber were used to validate the structural design itself. If this design is to be mass-produced in the future, more consideration should be given to material selection. Meanwhile, our experiment in this study only considered the design and implementation of a single person and a single embedded system and has not yet been able to transmit navigation data from a mobile phone to the system through Bluetooth or Wi-Fi communication modes, thereby further enhancing the device’s information-sharing capabilities with other smart devices.

In the future, we hope that this device will enhance users’ environmental perception, thereby increasing their sense of security and improving their travel experience while alleviating situational anxiety for cyclists. This advancement is anticipated to foster a transition from energy-intensive travel modes to greener, more energy-efficient, and cleaner alternatives, thus contributing to the establishment of a green travel system and healthier cities.

The design philosophy and implementation strategy of the BIIH provide new ideas and methods for the development of other interactive wearable devices. Based on bionic design principles, the BIIH improves the specific stiffness of the device. By integrating multimodal sensing with intelligent interaction capabilities, the BIIH serves as a versatile platform for human–machine interaction, electronic devices, and sensor applications. Looking ahead, we plan to incorporate additional physiological data from cyclists, such as electroencephalogram (EEG), galvanic skin response (GSR), and heart rate, into the BIIH. These data will assist users in making real-time travel decisions. Real-time readings of EEG, GSR, and heart rate can reflect the physiological state of cyclists. EEG reveals comprehensive data on cognitive load, attention levels, and fatigue of cyclists. GSR can reflect the activity of sweat glands, emotional states, and stress. Heart rate data reflect the physiological load on the body. Integrating these data can help cyclists make personalized training decisions in an all-round way, such as whether to maintain cycling speed, increase or decrease cycling distance, and whether to adjust breathing. This can not only improve their physical and mental health in addition to recreational and commuting cycling but also enhance their well-being. Moreover, when the integrated analysis shows that the physiological state of cyclists is abnormal and may affect cycling safety, the system can issue an alert.

In summary, the BIIH represents a significant advancement in wearable technology. The BIIH, which incorporates bionic principles, offers a unique combination of functionality, adaptability, and user-friendliness. Its potential for application in other wearable devices underscores its importance as a new platform for human–machine interaction and electronic applications. Future research will continue to explore and refine this technology, aiming to address existing limitations and unlock new possibilities in wearable electronics.

## 6. Conclusions

This investigation has culminated in the successful design and development of the BIIH, an advanced apparatus predicated on biomimetic principles, with the explicit aim of alleviating psychological distress and enhancing safety for urban cyclists. By integrating the STM32-embedded development platform with an array of sophisticated multimodal sensors—including those for temperature and humidity, ultrasonic detection, ambient light, and voice interaction—the BIIH achieves real-time environmental perception and seamless human–machine interactivity. The empirical findings substantiate the helmet’s exemplary performance in terms of signal response sensitivity, reliability of environmental awareness, and interaction security, thereby corroborating its efficacy as a portable and user-centric solution.

In comparison to conventional helmets, the BIIH exhibits pronounced advantages across critical dimensions, notably in its capacity for enhanced environmental perception, provision of real-time feedback, and augmented structural rigidity derived from biomimetic design validation. These attributes collectively elevate the wearer’s sense of security while delivering a demonstrably more comfortable and stress-mitigated cycling experience. Notwithstanding these achievements, this study acknowledges certain limitations warranting further exploration. Specifically, the structural safety of the BIIH under conditions of elevated velocity and its durability over prolonged usage remain subjects for additional rigorous assessment. Future enhancements are envisaged to incorporate a broader spectrum of physiological data, such as EEG readings and heart rate metrics, to furnish cyclists with more comprehensive real-time decision-making capabilities.

As an innovative contribution to the domain of wearable technology, the BIIH emerges as a versatile platform for advancing human–machine interaction and sensor-based applications. Its potential to promote greener, safer, and healthier urban environments aligns synergistically with the broader objective of establishing sustainable transportation systems. Prospective research endeavors will focus on addressing the delineated limitations and expanding the device’s functional repertoire, thereby further unlocking its latent potential within the realm of intelligent wearable technologies.

## Figures and Tables

**Table 1:** Technical parameters of STM32 F103C8T6.

STM32 Specification Parameter	
Operating Voltage	2.0–3.6 V
Input Voltage	3.3 V
Micro Controller	ARM Cortex-M3
Input Voltage (limit)	2.0–3.6 V
Digital I/O Pins	37
Analog I/O Pins	10
PWM Pins	16
DC I/O Pin	20 mA
DC for 3.3 V Pin	20 mA
Flash Memory	64 KB
SRAM	20 KB
Clock Frequency	72 MHz
Length	52 mm
Width	21 mm
Weight	7 g

**Table 2:** Response time of the sensors.

Unit	Type	Model	Response Time
PCB	STM 32	F103C8T6	2–8 ms
Input Sensor	Thermo-hygrometer sensor	DHT11	3–7 ms
Input Sensor	Thermo-hygrometer sensor	DHT22	3–7 ms
Input Sensor	Thermon sensor	AS6221	2–6 ms
Input Sensor	Distance-measuring sensor	HC-SR04	1–4 ms
Input Sensor	Light sensor	TEMT6000	12–40 ms
Input Sensor	Light sensor	BH1750	12–18 ms
Input Sensor	Light sensor	Microbit	6–9 ms
Output module	Voice interaction module	ASR01	1–4 ms
Output module	LED SMD	WS2812	3–8 ms
Output module	OLED	12864	3–8 ms
Output module	Fans	-	3–8 ms

**Table 3:** Components of the BIIH [00001].

Unit	Type	Model	Price
PCB	STM32	F103C8T6	$7.00
LED	SMD	5050	$1.50
Input sensor	Temperature and humidity sensor	DTH22	$1.10
Output model	Light sensor	BH1750	$1.00
Input sensor	Voice module sensor	ASRpro	$2.00
Input sensor	Ultrasonic sensor	HC-SR04	$0.80
Output model	Fan module	5 cm	$4.00
Output model	LEDS + OLED	WS2812 + OLED12864	$7.00

**Table 4:** Temperature and humidity model comparison.

		DHT11	DHT22	AS6221
		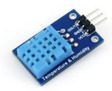	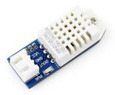	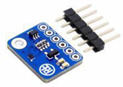
Temperature	Resolution	0.1 °C	0.1 °C	0.01 °C
	Accuracy	±2 °C	±0.5 °C	±0.09 °C
	Detection range	0~50 °C	−40~80 °C	0~42 °C
Humidity	Resolution	1% RH	0.1% RH	
	Accuracy	±5% RH (0~50 °C)	±2% RH (25 °C)	
	Detection range	0~99.9% RH	0~99.9% RH	
Operating voltage		3.3~5.5 V	3.3~5.5 V	3.5–5.0 V
Current supply		1~1.5 mA	1~1.5 mA	1~1.5 mA
Price		USD 1 to 5	USD 5 to 10	USD 4 to 7

**Table 5:** Comparison of light-intensive sensors.

Light-Intensive Sensor Comparison			
	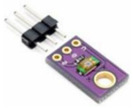	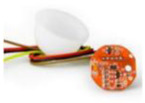	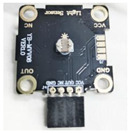
Operating voltage	1.5–6 V	1.8–4.5 V	3.3–5 V
Operating temperature	−10–100 °C	−10–85 °C	−10–70 °C
Power dissipation	100 mw	260 mw	200 mw
Sampling period	5 ms	20 ms	10 ms
Sampling range	0–10,200	1–100,000 lx	0.1–100,000 lx
Error range	2	10 lx	10 lx

**Table 6:** Distance-measuring module HC-SR04.

	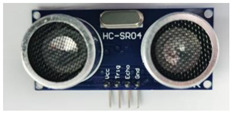	
Operating voltage	5	V
Operating current	15	mA
Measuring range	2–600	CM
Sensing Angle	<150	°
Operating temperature	−40 to 85	°C
Size	13.5	dm^3^

**Table 7:** Comparison of wind environment simulation data between BIIH and ordinary helmet.

Style	Wind Speed Range (m/s)	Range of Lift Coefficient (CL)	Range of Drag Coefficient (CD)	Range of Torque Coefficient (CM)
BIIH	0.5–10.5	0.10–15.70	0.50–15.00	0.00–3.10
Ordinary	0.5–10.5	0.10–27.60	0.50–20.00	0.00–9.60

**Table 8:** Experiments of call-words in different noise environment.

Categories	Options	Interviewees (N = 15)	Average Score (N = 5)
Satisfaction with Hazard Warning Function	Very satisfied (5)	12	4.66
	Satisfied (4)	1	
	Neutral (3)	2	
	Dissatisfied (2)	0	
	Very dissatisfied (1)	0	
Satisfaction with Temperature Regulation Function	Very satisfied (5)	13	4.80
	Satisfied (4)	1	
	Neutral (3)	1	
	Dissatisfied (2)	0	
	Very dissatisfied (1)	0	
Satisfaction with Brightness Function	Very satisfied (5)	11	4.40
	Satisfied (4)	1	
	Neutral (3)	2	
	Dissatisfied (2)	0	
	Very dissatisfied (1)	1	
Satisfaction with Mood-Soothing Function	Very satisfied (5)	14	4.86
	Satisfied (4)	0	
	Neutral (3)	1	
	Dissatisfied (2)	0	
	Very dissatisfied (1)	0	
Overall Satisfaction	Very satisfied (5)	12	4.73
	Satisfied (4)	2	
	Neutral (3)	1	
	Dissatisfied (2)	0	
	Very dissatisfied (1)	0	

## Data Availability

The data are unavailable due to privacy restrictions.

## Appendix A

We recruited 15 participants who reside in a Chinese megacity and exhibit significantly diverse physical characteristics and cycling habits. We also collected their basic demographic data to achieve a diverse sample population in terms of demographic features (as shown in Table A1). The data included the participants’ age, gender, frequency of cycling, cycling experience, and whether they owned their own helmet. These factors were selected because they have been identified as being closely related to perception of the cycling environment in the study by Patrick Rérat [84,85].

**Table A1:** Background information on the participants.

No.	Age	Gender	Frequency/M	Experience (Years)	Self-Owned Helmets
1	40s	Male	~2	35	No
2	30s	Female	~8	30	1
3	20s	Female	~10	5	1
4	20s	Male	~10	15	1
5	20s	Male	~20	10	1
6	20s	Male	~20	4	1
7	20s	Male	~20	12	1
8	40s	Female	~5	31	No
9	40s	Male	~20	30	No
10	50s	Male	~2	47	No
11	30s	Male	~30	33	3
12	40s	Female	~15	35	No
13	20s	Female	~10	5	1
14	20s	Female	~1	1	No
15	20s	Female	~5	5	No
